# What Is Inside the Sinus Tarsi? Mechanoreceptor Distribution, Typing and Clinical Relevance—A Histological and Immunohistochemical Synthesis

**DOI:** 10.3390/biomedicines13123052

**Published:** 2025-12-11

**Authors:** Alberto Arceri, Antonio Mazzotti, Gianmarco Di Paola, Federico Sgubbi, Laura Langone, Simone Ottavio Zielli, Francesca Veronesi, Gianluca Giavaresi, Paolo Mora, Cesare Faldini

**Affiliations:** 1 1st Orthopaedics and Traumatologic Clinic, IRCCS Istituto Ortopedico Rizzoli, 40136 Bologna, Italy; alberto.arceri@ior.it (A.A.); gianmarco.dipaola@ior.it (G.D.P.); federico.sgubbi@ior.it (F.S.); laura.langone@ior.it (L.L.); simoneottavio.zielli@ior.it (S.O.Z.); cesare.faldini@ior.it (C.F.); 2 Department of Biomedical and Neuromotor Sciences (DIBINEM), Alma Mater Studiorum University of Bologna, 40123 Bologna, Italy; paolo.mora@ior.it; 3 Surgical Sciences and Technologies, IRCCS Istituto Ortopedico Rizzoli, Via Di Barbiano 1/10, 40136 Bologna, Italy; francesca.veronesi@ior.it (F.V.); gianluca.giavaresi@ior.it (G.G.)

**Keywords:** sinus tarsi, mechanoreceptors, proprioception, immunohistochemistry, free nerve endings

## Abstract

The sinus tarsi, a small osseoligamentous recess of the subtalar joint, contains multiple soft-tissue structures with a complex sensory network. However, the detailed neural architecture and clinical significance of its innervation remain incompletely defined. The aim of this study was to conduct a comprehensive review of histological and immunohistochemical studies on the neural structures of the sinus tarsi. Histological staining and validated immunohistochemical markers (S100, p75, PGP9.5, neurofilament, myelin basic protein) were the methods used in these studies to analyze human sinus tarsi tissue. Across all investigations, free nerve endings predominated, while Ruffini, Pacinian and Golgi-like corpuscles were variably identified, mainly near ligament insertions. The sinus tarsi exhibits a dense and heterogeneous neural network that likely contributes to both pain perception and sensorimotor control. Further standardized and quantitative research is warranted to clarify the neurofunctional role of this region.

## 1. Introduction

Mechanoreceptors are responsible for the function of biological transducers, which are capable of converting mechanical stress into nerve impulses. It has been established that these receptors are present in several joints [1,2,3,4]. All the components of joint tissues (ligaments, joint capsule, fat pads, periarticular connective tissue) contain several mechanoreceptor types, each with distinctive morphology, adaptation behavior, stimulus sensitivity and functional roles in proprioception and pain signaling [3]. A classification by Freeman and Wyke [5] recognized the receptors in four morpho-functional types:

Ruffini corpuscles (Type I): these are slowly-adapting, low-threshold receptors, often partially encapsulated, with arborizing nerve terminals. They respond to sustained stretch, joint pressure and are sensitive to joint angle, static joint position and changes in direction. They contribute important afferent feedback for joint stability, posture and fine position sense [3,4,6,7,8].

Pacinian corpuscles (Type II): encapsulated, lamellated corpuscles which are rapidly adapting. They are especially responsive to dynamic changes—vibration, acceleration-deceleration of joint movement and rapid mechanical changes rather than steady pressure. They contribute to sensed motion, detecting onset and cessation of movement and rapid mechanical transitions [3,4,6,7,8].

Golgi-like receptors (Type III): typically found in ligamentous tissue. They respond to tension, stretch and extremes of joint motion (high strain), providing feedback about ligament load and possibly contributing to protective reflexes to prevent overstretching or injury. Their adaptation tends to be slower and their threshold higher [3,4,6,7,8].

Free nerve endings (Type IV): unencapsulated, often polymodal; these nerve endings respond to mechanical deformation, noxious stimuli (pain), temperature and sometimes chemical signals, exhibiting a slow and sustained pattern of activation. In joints and ligaments, they are abundant and serve primarily nociceptive functions, alerting to damage, overstrain, or inflammation [3,4,8].

The sinus tarsi is an osseoligamentous complex recess located between the talus and calcaneus, bounded anteriorly by the talonavicular joint and posteriorly by the posterior facet of the subtalar joint, with medial extension into the tarsal canal. This region contains several critical structures, including the roots of the inferior extensor retinaculum (IER), the oblique talocalcaneal ligament, tarsal canal interosseus ligaments, the subtalar joint capsule, as well as vascular and adipose tissue [9]. Beyond its biomechanical relevance, it has been theorized that the sinus tarsi contributes to proprioception adapting the foot to the ground [10] and for its involvement in pain syndromes, most notably sinus tarsi syndrome, due to its rich sensory innervation within the tissues present in its recess [11]. Of these, periarticular white adipose tissue—especially its fibrous subtype [12,13], which is abundant in mechanically stressed regions—has been shown to be richly innervated, providing indeed afferent sensory input [14,15]. Additionally, the subtalar joint capsule and ligaments, particularly those in close proximity to the ligament insertion into the bone, are characterized by a high density of innervation [11,16].

Some studies have suggested that mechanoreceptors within the sinus tarsi may mediate proprioceptive signaling critical for joint position sense and reflex stabilization, while free nerve endings predominantly serve nociceptive functions [11,17]. 

Despite these insights, the detailed neural architecture of the sinus tarsi has remained only partially elucidated, and its exact role in sensorimotor integration and pathology continues to be a focus of ongoing research. This gap in knowledge has potential important clinical relevance, since afferent input from the sinus tarsi may contribute to persistent pain in sinus tarsi syndrome [11] and even to the mechanism of action underlying corrective procedures such as subtalar arthroeresis [18].

The purpose of this review is to synthesize the available histological and immunohistochemical evidence on the neural structures of the sinus tarsi, to clarify their distribution and characteristics, and to explore their potential clinical implications.

## 2. Materials and Methods

Given the complexity and inconsistency of this topic, with heterogeneous and widely dispersed evidence, a comprehensive review approach was selected to map the existing literature, in order to identify key concepts, theoretical frameworks, sources of evidence and research gaps. This method facilitates a broad examination of the literature, without the restrictions of a systematic review.

A comprehensive search of the existing literature pertaining to histological and immunohistochemical data regarding sinus tarsi innervation was performed across the following electronic databases: PubMed/Medline, Scopus, Cochrane and OpenGrey. The search strategy combined the following keywords: “sinus tarsi” AND “mechanoreceptors” OR “nerve endings” OR “proprioception”. All keywords were meticulously examined both individually and combined with their Medical Subject Headings (MeSH) terms, adapting to the specific syntax of each platform. The search included all studies published until 1st October 2025. Reference lists of relevant articles were also screened manually.

Studies were considered eligible if they investigated the sinus tarsi with specific reference to mechanoreceptors, nerve endings, or proprioception. Histological, anatomical, and observational designs, as well as English and Italian articles, were included. Clinical trial, case reports unrelated to mechanoreceptors and studies outside the scope of the sinus tarsi region, as well as review papers, were excluded. 

Data extracted included authorship, publication year, study design, sample size, tissue analyzed, histological and immunohistochemical method, quantitative data and types of neural elements identified, when available. 

A qualitative thematic approach was employed throughout the reporting and summarizing of results. This method uses identification of key themes found in the literature and summarizing results using a descriptive synthesis. For each study, numerical data were extracted where available and presented as descriptive statistics (mean, standard deviation and percentage).

**Figure 1:**
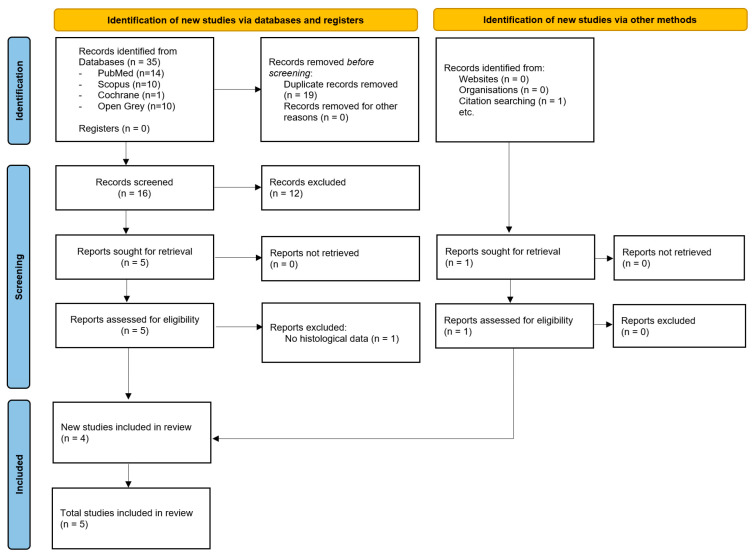
Flowchart of review process by PRISMA [19].

Although this work is not a systematic review, a PRISMA-style flow diagram (Figure 1) has been included to improve transparency and reproducibility in the literature selection process.

This diagram outlines the number of records that were identified, screened, assessed for eligibility and included in the final quantitative synthesis.

## 3. Tissue Analyzed

The extant literature includes studies that examined both cadaveric tissues [11,16,20] and surgical samples from living patients [17,21]. The tissue analyzed differed between studies, although the majority focused on fatty tissue [11,17,21]. Some studies examined the joint capsule of the sinus tarsi [11,17], while others analyzed ligaments [16,20,21] (Table 1).

## 4. Histological and Immunohistochemical Findings

### 4.1. Techniques for Neural Visualization

Akiyama et al. [17] utilized gold-chloride impregnation, effective for identifying neural elements but prone to non-specific staining of elastic fibers. In contrast, Rein et al. [11,16] applied immunohistochemistry with S100, p75 and PGP9.5, allowing precise neural localization, quantitative and qualitative assessment classified according to Freeman and Wyke [5]. In a similar manner, Spagnolli et al. [20] employed immuno-gold-silver staining with PGP9.5, S100 and NPY to detect neural fibers, but they did not perform a quantitative assessment. Morsy et al. [21] utilized an alternative examination method, employing M0762 for the analysis of neurofilament and A0623 for the assessment of myelin basic protein (Table 1).

Hematoxylin-eosin and Elastica-van Gieson complemented structural visualization [11,16,17,20,21] (Table 1).

### 4.2. Types and Distribution of Neural Elements

Data were organized into Table 1 which summarizes mechanoreceptor distribution. Across all studies, free nerve endings overwhelmingly dominate. A study by Rein et al. [11] reported markedly higher free-ending density in the subtalar capsule than in superficial fat pads; while no Pacinian corpuscles were detected in fat pads (Table 1). Another study by Rein et al. [16] reported that free nerve endings were present throughout all ligamentous samples. Ruffini endings were commonly identified, while Pacinian, Golgi-like corpuscles and unclassifiable corpuscles appeared less frequently (Table 1). Ratios confirmed the predominance of free endings (Table 1). Akiyama et al. [17] reported abundant free endings and occasional Pacinian, Ruffini and Golgi corpuscles in synovial tissue, with qualitative emphasis on nociceptive density. Ruffini and Golgi corpuscles were also reported by Spagnolli et al. [20] but they did not include any numerical data. Morsy et al. [21] described the presence of two types of nerve fibers: myelinated and unmyelinated.

### 4.3. Spatial and Neurovascular Correlates

Free endings cluster in synovial and subsynovial layers and between adipocytes of the sinus tarsi fat pads [11]. Conversely, mechanoreceptors are predominantly distributed near ligamentous insertions [16,20] (Table 1).

Both Rein studies [11,16] documented a strong positive correlation between free nerve endings and blood vessels (Spearman *r* = 0.73, *P* < 0.0001 in fat/capsule; *r* ≈ 0.57 in ligaments). 

## 5. Discussion

This review confirmed that the sinus tarsi contains a dense network of neural structures, dominated by free nerve endings while mechanoreceptors, such as Ruffini, Pacinian and Golgi-like corpuscles, were variably present. The cumulative evidence delineates the sinus tarsi as a highly innervated region integrating nociceptive and proprioceptive functions. In particular the consistent presence of free nerve endings supports its role in pain transmission, which likely could explain the clinical tenderness in sinus tarsi syndrome. Simultaneously, the presence of mechanoreceptors underscores a proprioceptive contribution to subtalar joint stability [14,15,16,18,22].

Regarding the immunohistochemical technique for neural visualization, the earliest investigation by Akiyama et al. [17] employed gold-chloride impregnation, a classical but non-specific method that stains not only nerve tissue but also blood vessels and reticular fibers, thus providing less specific imaging of neural elements in tissue. Although effective in revealing general neural distribution, this approach may overestimate innervation due to staining artifacts. The advent of immunohistochemistry marked a methodological turning point. By using specific neural markers such as S100, p75 and PGP9.5, Rein and colleagues [16] were able to delineate nerve fibers with high specificity, enabling both qualitative classification and quantitative measurement of mechanoreceptors. Similarly, Spagnolli et al. [20] utilized immune-gold-silver staining targeting PGP9.5, S100 and neuropeptide Y (NPY), confirming neural presence within the sinus tarsi but without providing quantitative data—thus limiting interpretability. The more recent study by Morsy et al. [21] expanded the technical repertoire through antibodies M0762 and A0623, directed against neurofilament and myelin basic protein, respectively. This dual staining approach differentiated myelinated from unmyelinated fibers, highlighting the heterogeneity of neural composition and supporting the concept of a complex, sensorimotor microenvironment.

Differences in mechanoreceptor prevalence and distribution across studies may reflect sampling (synovium vs. adipose vs ligament), methodological variability (staining and immunohistochemical markers) and tissue health (pathologic vs. cadaveric). However, across all studies, free nerve endings emerged as the predominant neural element, consistently present in all tissue layers examined. The Ruffini corpuscles were the second most common receptor type and were particularly concentrated at ligament insertions, where they may sense tension and angular displacement. Pacinian and Golgi-like corpuscles, although less frequent, were distributed near capsular and ligamentous attachments, consistent with their role in detecting rapid or high-amplitude joint movements. As previously described by Viladotet et al. [23], the ligaments of the sinus tarsi do not serve purely mechanical purposes. Rather, the presence of a multitude of receptor types within this region enables continuous sensorimotor monitoring of subtalar joint activity. The coexistence of mechanoreceptors with distinct physiological properties allows them to function in a complementary manner, ensuring precise sensorimotor control during complex joint movements. This ligament–bone interface therefore exhibits optimal anatomical and physiological characteristics for joint-protective sensorimotor functions, contributing to the maintenance of upright posture and the automatic coordination of gait. Based on this, the authors hypothesized that the subtalar joint acts as a neural mechanism that is responsible for the correction and fine adjustment of gait dynamics.

The study by Rein et al. [11] further established a strong neurovascular correlation, demonstrating that regions with richer capillary networks also exhibited higher neural density. This close anatomical relationship implies a neurovascular unit that may facilitate metabolic support and enhance neuronal responsiveness. Morsy et al. [21] extended this concept by highlighting the co-distribution of neural fibers and elastic elements, suggesting that neural activation may be coupled to the elastic recoil of the sinus tarsi during subtalar motion. They proposed that the sinus tarsi behaves as a “neurosensitive elastic organ.” This model implies that mechanical deformation of the elastic matrix may generate neural feedback signals contributing to postural control and joint alignment.

This sensory role has clinical relevance. Disorders involving hindfoot malalignment, such as pes planovalgus, may reflect or contribute to altered proprioceptive input within the sinus tarsi. However, to date, no studies have directly examined mechanoreceptor distribution in patients with flatfoot deformity, leaving this association unconfirmed but worthy of investigation. From a therapeutic perspective, the neurosensory function of the sinus tarsi could also play a role in the correction of flatfoot deformity, particularly in subtalar arthroeresis procedures for pediatric flatfoot. In detail, the recommended surgical intervention for growing-age flatfoot is subtalar arthroereisis, a procedure designed to restrict movement within the subtalar joint, thus allowing normal subtalar joint motion but preventing excessive hindfoot valgus [24,25]. While the mechanical effect of these implants is well-documented and often effective [25,26], Pisani postulated that correction of flexible flatfoot occurs not by mechanical constraint, but via proprioceptive reprogramming induced by stimulation within the sinus tarsi. This concept is referred to as “informative risi” [18]. In particular, Pisani [22] conducted a comparative study on fourteen patients, each treated with a classic endorthesis in one foot and a biofeedback arthroeresis in the contralateral foot, with the latter based on proprioceptive stimulation of the sinus tarsi nerve endings. He observed comparable clinical outcomes between the two procedures, thereby reinforcing the hypothesis that stimulation of neural structures within the sinus tarsi triggers a neuromotor adaptive response leading to gradual correction of deformity, so-called “biofeedback arthroeresis”. The hypothesis that subtalar implants exert part of their corrective effect via neurosensory modulation is intriguing and consistent with the available histological evidence. However, current clinical evidence is limited. The effects of implants are probably multifactorial, pending prospective studies that correlate histology, intraoperative/biomechanical measures and clinical outcomes.

Moreover, from a study [16] it emerged that mechanoreceptor density was reduced with aging (observed for Ruffini and unclassifiable corpuscles). This finding may compromise sensorimotor feedback, reinforcing the need for proprioceptive rehabilitation in chronic instability.

A study by Rein et al. [16] reported the presence of unclassifiable corpuscles in 68% of the analyzed ligamentous samples. However, the innate function and physiological properties of these corpuscles remain uncertain. Even advanced imaging techniques such as immunofluorescence and three-dimensional reconstruction of sensory nerve endings have revealed a substantial proportion of unclassifiable corpuscles in other joint structures [27,28,29]. This observation suggests an inherent limitation in the traditional Freeman and Wyke classification system [5], although in its modified form proposed by Hagert [30] the unclassifiable corpuscles were described, their exact role in joint proprioception remains unknown, underscoring the complexity and morphological variability of peripheral sensory endings in ligamentous tissues. These corpuscles are characterized by variable size, appearance and degree of encapsulation. Their considerable prevalence suggests that they may hold functional significance that is not yet fully understood. These structures could represent specialized or transitional mechanosensory units with functions that extend beyond the classical framework established by Freeman and Wyke. Given that they constitute a substantial proportion of the total mechanoreceptor population, it is reasonable to hypothesize that these corpuscles contribute to proprioceptive signaling within the sinus tarsi, possibly mediating complex or integrative forms of sensory input. While definitive evidence is lacking, their presence supports the long-standing hypotheses advanced by several authors [18,20,21,22].

There are some limitations. The selected studies exhibited an overall medium-low level of scientific evidence. Most studies were descriptive and lacked standardized quantification preventing robust statistical inference. Additionally, the included studies comprised different tissue samples—cadaveric and surgically excised tissue—which are not directly comparable. Sample sizes were small and heterogeneous, and demographic details (age, sex and comorbidities) were inconsistently reported. Furthermore, the absence of control specimens in surgical studies, as well as differences in measurement units and staining methods, limited generalizability. Future investigations should adopt standardized IHC protocols, include age- and pathology-stratified cohorts and integrate morphometric quantification.

## 6. Conclusions

The sinus tarsi contains a dense and heterogeneous neural network dominated by free nerve endings and by varying mechanoreceptor types and vascular structures. The quantification of immunohistochemistry data emphasizes spatial variation, with the most significant distribution observed at the level of synovial and ligament tissues and, to a lesser extent, fat tissue.

Based on these findings, the sinus tarsi may contribute to both nociceptive signaling and proprioceptive modulation. Clinical implications remain suggestive rather than conclusive and require further validation.

Future research should integrate histological mapping with functional studies to elucidate the exact neurophysiological mechanisms linking sinus tarsi innervation to foot biomechanics and surgical outcomes. 

## Figures and Tables

**Table 1:** Summary of the literature studies.

Study (Year)	Design—Sample Size	Age (y)	Tissue Analyzed	Methods/Stains Used	Mechanoreceptor Types Observed	Quantitative Data/Distribution (Means ± SD/Prevalence)
Akiyama et al., 1999 [17]	20 patients with sinus tarsi syndrome; 2 controls	25 (10–57)	Sinus tarsi’s synovial membrane and fatty tissue	Modified Gairns gold-chloride staining (15 specimens) + H&E (5 specimens)	Free nerve endings (predominant); Pacini, Golgi, Ruffini corpuscles.	Neural elements present in both symptomatic and control specimens, mainly in the synovial tissue. (No numeric counts reported)
Spagnolli et al., 2001 [20]	Cadaver study	Not reported	Dorsal talonavicular and plantar calcaneonavicular ligaments	Histological: H&E Immunohistochemistry: Immuno-gold-silver staining with PGP9.5, S100, NPY	Isolate and noradrenergic neural fibers, Ruffini and Golgi corpuscles.	No numeric counts reported. Mechanoreceptors mainly near bone insertions.
Rein et al., 2014 [11]	Cadaver study—13 feet	58 ± 16	Three specimen types from sinus-tarsi region: superficial fat pad at IER, deep fat pad at IER and subtalar joint capsule (inside sinus tarsi)	*Histological:* H&E, Elastica van Gieson (EvG); *Immunohistochemistry*: S100, p75, PGP9.5; vascular marker sm-actin.	Free nerve endings (predominant) among adipocytes and in subsynovial layer. Ruffini and Golgi-like rare. No Pacini corpuscles detected.	Free nerve endings—subtalar joint capsule: 88.8 ± 71.3 cm^2^; superficial fat pad (IER): 47.8 ± 31.3 cm^2^ (*p* < 0.001). No Pacini found
Rein et al., 2013 [16]	Cadaver study—140 ligaments (from 10 feet)	58 ± 20	Ankle ligaments, grouped into complexes: lateral, medial, sinus tarsi complex, ATiFL (syndesmosis)	H&E + *immunohistochemistry*: S100, p75, PGP9.5; vascular marker sm-actin.	Free nerve endings (present in 100% of ligaments), Ruffini, Pacini, Golgi-like, unclassifiable corpuscles.	Free nerve endings 100%; Ruffini endings in 108/140 (77%); Pacini in 49/140 (35%); Golgi-like in 15/140 (11%); unclassifiable corpuscles in 95/140 (68%). Ratios (mean across all ligaments): Ruffini:Pacini 4.7:1; Free:Ruffini 122:1; Free:Pacini 582:1; Free:Golgi 1890:1. Free endings were more abundant in epiligamentous and near bone insertions.
Morsy and Filler, 2017 [21]	46 surgical cases	39 (8–77)	Soft tissue material from the sinus tarsi	H&E, Trichrome, Orcein. *Immunohistochemistry:* M0762 for neurofilament, A0623 for Myelin basic protein	Myelinated and unmyelinated fibers	No numeric counts reported

*Abbreviation*: H&E Haematoxylin-Eosin, ATiFL anterior tibiofibular ligament, IER inferior extensor retinaculum.

## Data Availability

The data presented in this study are available on request from the corresponding author; however, restrictions apply to the availability of these data, which were used under license for this study, and they are thus not publicly available.
